# Caesarean section and respiratory system disorders in newborns

**DOI:** 10.1016/j.eurox.2024.100336

**Published:** 2024-08-10

**Authors:** Maryam Yeganegi, Reza Bahrami, Sepideh Azizi, Zahra Marzbanrad, Nazanin Hajizadeh, Seyed Reza Mirjalili, Maryam Saeida-Ardekani, Mohamad Hosein Lookzadeh, Kamran Alijanpour, Maryam Aghasipour, Mohammad Golshan-Tafti, Mahmood Noorishadkam, Hossein Neamatzadeh

**Affiliations:** a Department of Obstetrics and Gynecology, Iranshahr University of Medical Sciences, Iranshahr, Iran; b Neonatal Research Center, Shiraz University of Medical Sciences, Shiraz, Iran; c Shahid Akbarabadi Clinical Research Development Unit, Iran University of Medical Sciences, Tehran, Iran; d Department of Obstetrics and Gynecology, Firoozgar Clinical Research Development Center, Firoozgar Hospital, Iran University of Medical Sciences, Tehran, Iran; e Prevention Gynecology Research Center, Imam Hossein hospital, Shahid Beheshti University of Medical Sciences, Tehran, Iran; f Mother and Newborn Health Research Center, Shahid Sadoughi University of Medical Sciences, Yazd, Iran; g General Practitioner, Babol University of Medical Sciences, Babol, Iran; h Department of Cancer Biology, College of Medicine, University of Cincinnati, Cincinnati, OH, USA; i Department of Pediatrics, Islamic Azad University of Yazd, Yazd, Iran

**Keywords:** Cesarean section, Respiratory System, Transient Tachypnea, Persistent Pulmonary Hypertension

## Abstract

Cesarean section (C-section) delivery is associated with a higher risk of respiratory problems in newborns, particularly if performed electively at 37 weeks. This risk is greater than with spontaneous or induced labor but diminishes as gestation advances. To lower the incidence of respiratory issues in newborns, it is vital to promote natural labor, avoid unnecessary C-sections, and offer thorough prenatal care. Healthcare providers and expectant mothers should assess the risks and benefits of elective C-sections carefully. By advocating for natural labor and reducing unnecessary C-sections, the occurrence of respiratory problems in newborns can be decreased. Adequate prenatal care and monitoring are crucial for identifying and managing potential risk factors for respiratory diseases in newborns. It is crucial for healthcare professionals to educate expectant mothers about the risks of elective C-sections and the advantages of allowing labor to progress naturally. By fostering transparent communication and collaborative decision-making between healthcare providers and pregnant women, well-informed choices can be made that prioritize the health of both the mother and the baby. Furthermore, ongoing research and advancements in medical technology can improve our understanding of how delivery methods affect newborn respiratory health, ultimately leading to better outcomes and care practices in the future.

## Introduction

Since the 1990s, there has been a notable increase in cesarean section (C-section) rates due to advancements in medical standards and related socio-cultural factors. This rise has raised concerns about potential overuse of the procedure and its associated risks [1]. Critics argue that some C-sections may be performed unnecessarily, exposing women and their babies to avoidable health risks. Additionally, the increase in C-section rates has sparked discussions about the impact on healthcare costs and resources [1], [2]. Ongoing research and efforts are focused on understanding the factors driving the increase in C-section rates and developing guidelines for appropriate use of the procedure. The World Health Organization (WHO) has noted a troubling global rise in C-section rates, which have climbed from about 7 % in 1990 to 21 % in 2015, surpassing the WHO's recommended ideal rate of 10 % to 15 % [1]. This increase is concerning because not all C-section procedures are medically necessary, with a growing number of nonmedically indicated C-sections and "caesarean on maternal request". In at least 15 countries, the C-section rate exceeds 40 %, including the Dominican Republic, Brazil, Egypt, and Turkey [3], [4], [5]. Factors contributing to the rising C-section rates include previous C-section, labor dystocia, indeterminate fetal heart rate tracing, suspected fetal macrosomia, malposition, risk-adapted obstetrics, litigation fears, and elective cesarean in educated women[6]. Despite this sharp rise, overall improvements in neonatal outcomes have been limited. Specifically, considering the respiratory, immune, and neuropsychiatric systems, it is evident that C-sections, especially elective ones, have noticeable short-term effects on newborns and also influence their long-term prognosis [7], [8], [9].

C-section has both short-term and long-term effects on newborns. In the short term, it disrupts the establishment of gut microbiota, resulting in a microbiome that resembles the environment and the mother's skin rather than the vaginal microbiome. This change can increase the risk of early life infections and non-transmissible diseases such as inflammatory diseases, allergies, and metabolic diseases. In the long term, children born by C-section have a higher risk of developing asthma, wheezing, and respiratory infections [9]. Additionally, it is associated with an increased risk of neonatal ICU admission, respiratory complications, and low 5-minute APGAR scores. C-section also negatively impacts breastfeeding practices, leading to less skin-to-skin contact and delayed initiation of breastfeeding [4]. These findings underscore the importance of considering the potential risks and benefits of C-section delivery for both short-term and long-term newborn health outcomes. While C-section alone may not directly cause neonatal diseases, premature infants face a higher risk of illness compared to full-term infants [4], [10]. Moreover, elective early cesarean deliveries have contributed to a rise in the birth rate of late preterm infants, significantly impacting neonatal morbidity and mortality [11]. A retrospective study involving 2228 newborns revealed that infants delivered via cesarean at 35, 36, and 37 weeks faced a 10-fold, 2-fold, and 1-fold increased risk of severe adverse conditions, respectively [12]. Furthermore, researchers have confirmed that cesarean deliveries performed after the onset of uterine contractions and after 39 weeks can lower neonatal mortality [13], [14]. As a result, the American College of Obstetricians and Gynecologists (ACOG) recommends that all elective C-sections be scheduled after 39 weeks of gestation, particularly after the onset of contractions and once lung maturity has been established [13]. While C-section can lower the risk of intrauterine distress and meconium aspiration, numerous studies both domestically and internationally have consistently shown that babies delivered via C-section have a higher likelihood of respiratory diseases compared to those born vaginally [6], [8], [13]. This risk escalates with fetal age, with a gradual decrease as age increases. Additionally, the incidence of respiratory diseases in infants born via C-section at 37 weeks is three times higher than those born at 39 to 40 weeks [13].

Prematurity, meconium-stained amniotic fluid, obstructed parturition, and C-section are significant factors contributing to respiratory distress in neonates [15], [16]. The onset of respiratory conditions in neonates can be attributed to perinatal hypoxia, pulmonary immaturity and liquid absorption, pulmonary circulation complications, infections, and congenital abnormalities of the airway and lungs [17], [18]. The prevalence of respiratory distress is higher in neonates delivered via C-section compared to those delivered vaginally due to the absence of lung compression during delivery. The probability of respiratory complications in neonates delivered via elective C-section varies from 2.8 % to 27.1 % depending on the gestational age [8], [19], [20]. Elective C-sections performed between 35 and 38 weeks of gestation have been associated with respiratory morbidity in newborns [7], [12], [21], [22], [23]. The most commonly observed respiratory disorders in neonates admitted to the neonatal intensive care unit (NICU) include transient tachypnea of the newborn (TTN) and respiratory distress syndrome (RDS) [7], [16]. Administering antenatal corticosteroids in elective C-sections has been proposed as a potential preventive intervention to mitigate the risk and severity of respiratory distress in newborns. However, further investigation is required to ascertain the efficacy of prophylactic corticosteroids in reducing respiratory morbidity in neonates delivered via C-section [21].

We conducted a thorough review of previous scholarly works on the link between cesarean section and respiratory system diseases in newborns. The lack of exposure to the mother's vaginal and fecal microbiota is a key factor in this relationship, as they are essential for the development of the infant's immune system and respiratory health. Furthermore, the stress of labor and hormonal changes during vaginal delivery are thought to have a protective effect on the infant's respiratory system[4], [24]. Additional research is necessary to fully understand the intricate interplay between cesarean section and respiratory system diseases in newborns.

## Neonatal respiratory distress syndrome

C-section has been associated with an increased risk of adverse respiratory outcomes in infants, particularly when delivery occurs before the onset of labor [25]. Studies have consistently shown a higher risk of neonatal respiratory morbidity in infants born by elective C-section, especially at 37 and 38 weeks' gestation [8]. However, performing elective C-section in the week 39 + 0 to 39 + 6 of pregnancy has been linked to a significant reduction in neonatal respiratory morbidity [26], [27]. While elective C-section reduces the occurrence of birth asphyxia and trauma, it increases the risk of respiratory distress secondary to TTN, surfactant deficiency, and pulmonary hypertension [20]. Additionally, infants born to obese mothers and delivered by C-section are at a higher risk of developing TTN and respiratory distress syndrome [28]. Neonatal respiratory distress syndrome (NRDS) mainly occurs due to decreased synthesis and secretion of pulmonary surfactant, with NRDS incidence decreasing as gestational age increases. Infants born through elective C-section before full term are at a significant risk of respiratory problems. Even babies born through elective C-section at term without corticosteroid cover face a risk of RDS. Antenatal dexamethasone use in late preterm and term pregnancies aims to improve neonatal morbidity and mortality, highlighting C-section as a risk factor for neonatal respiratory distress [29]. Furthermore, elective C-section at term has been associated with an increased likelihood of respiratory distress at birth in neonates [30]. Infants born by elective C-section are predisposed to neonatal respiratory disorders, such as TTN and respiratory distress syndrome [31]. Moreover, a high incidence of respiratory distress and neonatal intensive care unit admissions has been documented for infants delivered by C-section before the onset of spontaneous labor [32]. Finally, elective C-section has been associated with a higher risk of iatrogenic respiratory distress syndrome for the neonate [33].

Several studies have indicated that C-section significantly increases the risk of NRDS compared to vaginal delivery. A study in India found that 65 % of the infants in the research were delivered by C-section. Additionally, 27.8 % of these infants experienced RDS [34]. In another study, the risk factors for RDS were compared between early-term (37–38 weeks) and full-term (39–40 weeks) births, revealing that C-section posed a risk for RDS in both groups [35]. In 2024, Ajmal et al. conducted a retrospective cohort study with 6316 women in Qatar using data from a national perinatal database obtained from a single tertiary maternity care hospital. The study found that the risk of RDS (RR = 1.5, 95 % CI 1.2–2.0, p = 0.002) and NICU admissions (RR = 1.3, 95 % CI 1.0–1.6, p = 0.038) in neonates born via multiple cesarean deliveries is higher with lower gestational age and birthweight in these groups[36]. In another Arabian country, Al Riyami et al. found that the rate of neonatal RDS among Omani women (n = 650) who underwent elective C-section at Sultan Qaboos University Hospital over a five-year period was 2.5 %[11]. A retrospective study in Zhengzhou demonstrated a strong correlation between the occurrence of RDS in term infants (37–42 weeks) and elective C-section and pneumonia without the onset of labor[37]. In 2019, Li and colleagues analyzed 26 studies and found that neonatal RDS was significantly linked to C-section, with an odds ratio of 1.76 (95 % CI 1.48–2.09). When they looked at subgroups by continent, they discovered that the combined odds ratio for neonatal RDS was 1.76 (95 % CI 1.45–2.13) in North America and 1.79 (95 % CI 1.18–2.71) in other continents. Furthermore, their research showed that the risk of neonatal RDS associated with elective C-section was 2.38 (95 % CI 1.89–2.99) [38].

The development of RDS after a C-section involves two main aspects. Firstly, it includes inadequate secretion or malfunction of pulmonary surfactant and impaired clearance of pulmonary fluid in premature delivery. Recent research has shown the critical role of vaginal squeezing during natural childbirth in facilitating lung fluid clearance [15], [35]. Furthermore, the amiloride-sensitive sodium channel, also known as the epithelial sodium channel (ENaC), in alveolar surface epithelial cells significantly impacts lung fluid clearance, thus playing a major role in RDS development [39]. Secondly, the absence of squeezing and hypoxic stimulation from natural childbirth, along with the use of narcotics and sedatives, leads to a delay in the initiation of newborn breathing and a reduction in the levels of relevant hormones, particularly catecholamines and glucocorticoids. This delay further hampers the clearance of lung fluid and the establishment of normal breathing[29], [40]. Additionally, most infants undergoing C-section will have a history of intrauterine distress and asphyxia [41]. Lastly, it is important to highlight that respiratory distress symptoms in near-term infants may manifest late and might not be readily apparent, posing significant challenges in terms of diagnosis and treatment [42]. The potential complications arising from RDS after a C-section highlight the need for close monitoring and immediate intervention to support the respiratory function of the newborn [43]. This may involve the administration of exogenous surfactant, mechanical ventilation, and other respiratory support measures [43]. Additionally, healthcare providers should be vigilant in assessing and addressing any signs of respiratory distress in infants born via C-section, particularly those born prematurely or with a history of intrauterine distress. Early detection and management of RDS are crucial in improving outcomes and reducing the risk of long-term respiratory complications [44], [45].

## Transient tachypnea of the newborn (TTN)

TTN is a common respiratory disorder characterized by tachypnea and respiratory distress shortly after birth [44], [45]. TTN is a self-limiting condition that occurs shortly after birth and is characterized by mild to moderate distress. Most cases of TTN have mild respiratory distress and symptoms typically resolve within a few days. It is associated with delayed lung fluid clearance, often due to inadequate respiratory epithelial sodium ion transport and lung fluid reabsorption [46]. The condition is frequently observed in term or near-term infants [47]. Infants born to obese and diabetic mothers and delivered by C-section are at a higher risk of TTN, respiratory distress syndrome, and hypoglycemia in the early neonatal period [28]. Additionally, TTN has been linked to elective C-section before 39 weeks of gestation, with an increased risk of neonatal morbidities [25]. C-section delivery is associated with TTN, characterized by delayed resorption of lung fluid and can lead to respiratory distress in newborns. Lung function tests revealed that babies delivered by C-section have an excessive amount of lung fluid, leading to lower thoracic gas volume compared to babies delivered vaginally. Lung ultrasound studies have also shown higher levels of lung liquid in C-section babies compared to those born vaginally. Early identification of infants at risk for pulmonary maladaptation through noninvasive bedside ultrasound can assist in the management of TTN. Antenatal corticosteroid use has been studied as a potential intervention to reduce the incidence of TTN and other neonatal morbidities associated with elective C-section [29]. The type of anesthesia used during C-section has also been examined in relation to the occurrence of TTN, particularly general or combined epidural-spinal anesthesia [48]. Furthermore, the timing of planned C-section has been linked to neonatal outcomes, including TTN, suggesting that elective C-section before 39 weeks of gestation may elevate the risk of TTN and other respiratory morbidities [25], [49]. Furthermore, the duration from uterine incision to fetal delivery during C-section has been linked to neonatal outcomes, including TTN [50].

TTN incidence declines as gestation age increases, affecting around 10 % of infants born at 33–34 weeks, 5 % at 35–36 weeks, and less than 1 % at full term [51]. Maternal risk factors include early delivery before 39 weeks, C-section without labor, gestational diabetes, and maternal asthma. Fetal risk factors include male gender, perinatal asphyxia, prematurity, and small and large for gestational age infants [51]. A study by Özdoğar et al. revealed that the incidence of TTN was 2.9 % in vaginal deliveries and 8.5 % in C-sections in İzmir, Turkey [36]. Chavan et al. reported that the incidence of TTN was 16 per 1000 live births, with 63.5 % being male, 75.7 % term births, 70.3 % delivered via lower section C-section (LSCS), and 66.2 % normal birth weight (≥2.5 kg) infants in Pune, India [52]. Furthermore, it was mentioned that the risk of TTN increases 2 to 6 times in babies born via elective C-section compared to those born with normal delivery [53]. However, an Egyptian study by Elgarhy et al. did not find a significant difference in the occurrence of TTN between the study group (given Misoprostol) and the control group (given placebo) in women undergoing C-section [54]. In a case-control study of 200 newborns with respiratory distress at the NICU in AL-Kansaa Teaching Hospital in Mosul from September 1st, 2011 to September 1st, 2013, Hamdoon et al. found that TTN is strongly associated with elective C-section, male sex, late prematurity, maternal diabetes, maternal asthma, birth asphyxia, low birth weight (1500–2500 g), prolonged labor, use of forceps or vacuum, and in vitro fertilization [55]. Tutdibi et al. conducted a retrospective analysis of 13,346 newborns over 37 weeks of age and found that the prevalence of TTN in elective C-section without uterine contractions decreased with increasing gestational age compared to vaginal delivery [56]. Children with TTN require sustained oxygen support and are more likely to experience complications such as air leakage and persistent pulmonary hypertension (PPHN) [56]. Altman et al. analyzed 471,194 newborns aged 30 to 37 weeks and identified risk factors for TTN, including elective C-section, male gender, low 5-minute Apgar score, and low gestational age [57].

The potential development of TTN following C-section involves several factors: (1) During the fetal stage, the expression of epithelial sodium channels (ENaC) decreases as gestational age increases, leading to reduced reabsorption of pulmonary fluid. Infants delivered via C-section before 35 weeks of gestation are more susceptible to TTN due to this phenomenon. (2) Incomplete pulmonary development and inadequate synthesis of alveolar surfactant can hinder the reabsorption of lung fluid. (3) The absence of the stress induced by labor and premature birth can lead to lower hormone levels in newborns, delaying the elimination of pulmonary fluid. (4) Asphyxia and distress before C-section can lead to the aspiration of amniotic fluid and an increase in lung fluid. Severe hypoxia and increased vascular permeability due to inflammation significantly increase the risk of TTN. (5) Mothers who undergo C-section often have underlying conditions such as pregnancy-induced hypertension, which can increase the production of pulmonary fluid in offspring. Additionally, the use of sedative anesthetics during C-section can affect alveolar dilation and pulmonary blood vessels, thereby impacting the production, absorption, and clearance of pulmonary fluid. Some studies suggest that newborns of mothers with a history of asthma are more vulnerable to TTN, possibly due to changes in their mothers' response to catecholamines. Infants with TTN may subsequently develop wheezing symptoms, thereby increasing the likelihood of conditions such as bronchiolitis, asthma, acute bronchitis, and chronic bronchitis [58], [59].

## Persistent pulmonary hypertension of the newborn (PPHN)

PPHN is a condition characterized by failure of pulmonary vascular resistance to decrease at birth, leading to decreased pulmonary blood flow and shunting of unoxygenated blood to the systemic circulation. Prenatal exposure to antidepressants has been associated with PPHN, and the mode of delivery, particularly C-section, has been found to be more common in infants with PPHN, considered a risk factor for the condition [60]. Additionally, planned elective C-section at term has been identified as the main risk factor for pulmonary fluid resorption disorders, especially if performed before labor and at terms less than 38 weeks [61]. The prevalence of repeat C-section in a tertiary care hospital has been noted to be quite high, indicating a significant number of women undergoing multiple cesarean deliveries [62]. Furthermore, the quality of discharge teaching has been found to impact women's discharge readiness and post-C-section outcomes, highlighting the importance of postoperative care and education for women undergoing C-section [63]. C-section delivery is estimated to be associated with a five-fold higher risk of PPHN compared to vaginal delivery. However, the mechanisms underlying this association are still poorly understood. Factors such as iatrogenic prematurity, higher rates of late preterm delivery, lack of physiological changes during labor, limited endogenous pulmonary vasodilator synthesis, lower levels of protective antioxidants, and increased risk of respiratory distress syndrome contribute to the link between C-section and PPHN [64]. Meconium aspiration syndrome, birth asphyxia, and respiratory distress syndrome are common postnatal causes of PPHN [65]. Lower levels of circulating norepinephrine in neonates after C-section may also contribute to the higher incidence of PPHN [66]. Preventive measures such as antenatal corticosteroids, accurate informed consent, delaying elective C-section, and maternal antioxidant supplementation may help mitigate the effects of C-section delivery and minimize C-section-related PPHN [64].

In a study by Arshad et al. in Multan, Pakistan, it was found that 57.4 % of PPHN cases were linked to C-section delivery [65]. Likewise, research by Nchabeleng et al. in Limpopo Province, South Africa, showed that 59 % of PPHN cases involved C-section delivery [67]. In a study by Winovitch et al., it was found that in a group of 300 newborns, the prevalence of PPHN was 0.69 %. The risk ratio (RR) for PPHN in cases of C-section without uterine contractions, compared to C-section with contractions, was 2.0 with a 95 % CI ranging from 1.3 to 3.1. Similarly, compared to natural vaginal delivery, the relative risk of PPHN was 3.4 (95 % CI 2.1–5.5). Furthermore, for the same delivery mode, the relative risk of PPHN was 3.7 (95 % CI 2.3–6.1) for births before 37 weeks of gestation, 2.2 (95 % CI 1.4–3.4) for births after 37 weeks of gestation, 3.4 (95 % CI 2.1–5.5) for birth weights below 2500 g, and 1.9 (95 % CI 1.3–3.0) for birth weights exceeding 2500 g [68].

Potential mechanisms of PPHN from C-section may be linked to various factors. Firstly, premature infants often face complications such as patent ductus arteriosus, a well-known high-risk factor for PPHN. In addition, C-section infants are susceptible to respiratory diseases, including RDS and TTN, which pose a significant risk for PPHN development [69]. This susceptibility can be attributed to the lack of stimulation during normal vaginal delivery, as well as the prematurity of these infants. Consequently, their hormonal levels related to PPHN, particularly glucocorticoid and catecholamine hormones, are noticeably lower compared to vaginally delivered infants [69]. Furthermore, the use of anesthesia and analgesia during C-section delivery can also impact the respiratory function of the infant, potentially leading to PPHN. The altered breathing patterns and reduced respiratory drive in the newborn can contribute to the development of this condition. Additionally, the presence of meconium aspiration syndrome in C-section infants further increases the risk of PPHN due to the potential damage to the pulmonary vasculature. Overall, the combination of prematurity, respiratory complications, and potential exposure to meconium during C-section delivery significantly heightens the likelihood of PPHN in these infants [70].

## Discussion

C-section delivery has been found to have various effects on newborns. Research indicates that elective C-sections carry a higher risk of negative perinatal outcomes and care practices compared to vaginal deliveries [71]. One study found that infants delivered through C-section had lower levels of zinc in their umbilical cords, potentially leading to decreased birth weight [72]. However, another study found that paternal skin-to-skin contact after C-section delivery had positive effects on newborns, such as stabilizing their physical conditions, increasing rates of breastfeeding, and reducing paternal anxiety and depression [73]. The effects of administering bupivacaine in combination with various doses of fentanyl during C-sections on newborns have not been explicitly outlined [74]. Additionally, refraining from oro-and naso-pharyngeal suction (ONPS) in newborns born via C-section has no significant impact on oxygen saturation (SpO2) and heart rate during the initial postpartum hour [75]. Multiple studies have shown a higher occurrence of respiratory distress and admissions to the NICU in infants delivered through C-section before spontaneous labor. However, elective C-section has been associated with a decreased likelihood of birth asphyxia, trauma, and meconium aspiration, as discussed in other healthcare literature[20], [32]. Accurately determining the prevalence of respiratory failure and long-term outcomes in full-term and near-term infants is challenging due to limited databases compared to premature infants. Nevertheless, it is estimated that a significant number of term infants delivered via C-section are annually admitted to neonatal intensive care units in the United States, with diagnoses including TTN and severe PPHN/hypoxic respiratory failure. Some reports also suggest increased rates of mechanical ventilation, oxygen therapy, extracorporeal membrane oxygenation (ECMO), and mortality. Studies by Madar et al. and Roth-Kleiner et al. have shown that infants experiencing respiratory distress following a C-section require significantly more mechanical ventilation [20].

The impact of C-section delivery on newborns' respiratory system diseases has been a topic of debate. Studies have highlighted a connection between C-section and a heightened risk of immune and metabolic disorders in infants [76]. Research has shown that non-medically necessary cesarean sections negatively affect newborns' health, leading to lower Apgar scores and a higher prevalence of respiratory issues in the neonatal period [77]. Elective cesarean sections have been associated with an increased risk of neonatal respiratory complications, such as respiratory distress syndrome and TTN, compared to vaginal delivery [78]. It has been suggested that the absence of lung compression during cesarean section and the lack of changes in the hormonal environment between the mother and fetus contribute to the heightened likelihood of respiratory morbidity in infants delivered via cesarean section [79]. The timing of elective cesarean sections is a critical factor, with deliveries at 37 weeks gestation linked to a greater risk of neonatal complications, including respiratory disorders [22]. The exact pathogenesis of this relationship remains uncertain, but hypotheses suggest that variances in bacterial colonization and maturation of the immune system may play a role. The risk factors and underlying mechanisms of childhood asthma, potentially linked to C-section, are not fully understood. Additionally, the use of prenatal betamethasone in pregnancies with elective cesarean sections at 38 weeks gestation has been examined for its impact on negative neonatal respiratory outcomes, emphasizing the importance of considering interventions to mitigate the risks associated with cesarean deliveries [80]. The microbiota of infants delivered via cesarean section differs from that of vaginally delivered infants, and efforts to restore the microbiota of cesarean-born infants have shown promising results in influencing the respiratory health of newborns [81]. A prospective study conducted by Salem et al. within the Bern Basel Infant Lung Development birth cohort, consisting of a cohort (n = 578) of unselected, healthy, full-term, Caucasian neonates, aimed to investigate the potential association between delivery method and alterations in respiratory and atopic outcomes during infancy and at school age. The authors compared weekly respiratory symptoms in the first year of life and pulmonary function in infants at 5 weeks of age between those delivered via cesarean section (N = 114) and those delivered vaginally (N = 464) after a full-term pregnancy in healthy females. The results indicated that C-section was not linked to respiratory symptoms during the first year of life or to various respiratory or atopic outcomes at school age when compared to vaginal delivery[24]. These findings suggest that C-section does not have enduring effects on the respiratory well-being of the child.

Delivery mode, whether vaginal or by C-section, does not appear to affect the occurrence of bronchopulmonary dysplasia (BPD). Opting for a C-section when a vaginal delivery is possible cannot be justified solely to reduce the incidence of BPD [82]. However, the first 10 min after birth are critical for the short and long-term outcomes of premature infants, including the risk of developing BPD [83]. Neonates who receive resuscitation with high levels of oxygen during this period are at higher risk of developing BPD [84]. Overall, there is insufficient evidence to support using cesarean section to reduce the risk of BPD in pregnancies where vaginal delivery is an option. Effective management of infants with evolving or established BPD involves respiratory support techniques and medical interventions [85]. Ventilation strategies for infants with severe BPD should consider the diverse nature of the disease and aim for a physiologically appropriate approach, such as using combined volume-guaranteed synchronized intermittent mechanical ventilation and pressure support ventilation [86]. Among healthy full-term infants, cesarean section delivery does not seem to impact respiratory symptoms, respiratory rate, or lung function in the first year of life [24]. The EPICE Cohort study, which included 7435 live births (24 +0 to 31 +6 weeks postmenstrual age) in 19 regions across 11 European countries, found that cesarean section delivery was not associated with BPD or the combined outcome of death or BPD compared to vaginal delivery. This remained consistent even after adjusting for perinatal and neonatal risk factors, and in pregnancies where vaginal delivery was an option [24]. Further analyses involving singletons, infants in cephalic presentation, and infants of ≥ 26 + 0 weeks of gestation did not change the results for BPD, severe BPD, and death or BPD, even in regions with a high cesarean section rate[24]. The study suggests that reducing BPD does not justify using cesarean section in pregnancies where vaginal delivery is possible.

## Conclusion

In essence, there is significant concern about neonatal respiratory complications in neonatal healthcare, especially in elective C-section. Factors such as maternal obesity, anesthesia type, and delivery timing are associated with conditions like NRDS, PPHN, and TTN. This underscores the need to carefully consider delivery method and timing to reduce the risk of pulmonary fluid resorption disorders in newborns. Research shows that C-section deliveries, especially without medical necessity and at earlier gestational stages, are linked to increased susceptibility to respiratory ailments in newborns. The lack of exposure to maternal microbiota during vaginal delivery and the physiological processes of labor may contribute to the higher incidence of respiratory issues in babies delivered via C-section. The prevalence of repeat C-sections and the impact of discharge teaching on post-C-section outcomes highlight the importance of providing comprehensive care for women undergoing C-section. Understanding the risk factors and potential interventions is crucial to improving neonatal outcomes and reducing respiratory complications in newborns.

## Ethics approval

This is a meta-analysis and ethical approval is not required.

## Funding

No funding is associated with the work presented in this article.

## CRediT authorship contribution statement

**Mohammad Golshan-Tafti:** Investigation, Supervision. **Mahmood Noorishadkam:** Supervision, Validation. **Reza Bahrami:** Conceptualization, Data curation. **Maryam Yeganegi:** Conceptualization, Data curation. **Maryam Saeida-Ardekani:** Writing – original draft, Writing – review & editing. **Mohamadhosein Lookzadeh:** Supervision, Validation. **Kamarn Alijanpour:** Resources. **Maryam Aghasipour:** Writing – original draft, Writing – review & editing. **Sepideh Azizi:** Investigation. **Zahra Marzbanrad:** Methodology, Project administration. **Nazanin Hajizadeh:** Investigation, Visualization. **Seyed Reza Mirjalili:** Validation. **Hossein Neamatzadeh:** Project administration, Writing – original draft, Writing – review & editing.

## Declaration of Competing Interest

The authors declare that they have no known competing financial interests or personal relationships that could have appeared to influence the work reported in this paper.

## Data Availability

The data supporting this study can be obtained from the corresponding author upon request.
